# The complete mitochondrial genome of the critically endangered Atlantic humpback dolphin, *Sousa teuszii* (Kükenthal, 1892)

**DOI:** 10.1080/23802359.2019.1700196

**Published:** 2019-12-13

**Authors:** Michael R. McGowen, Katherine R. Murphy, Ibrahima Ndong, Charles W. Potter, Lucy W. Keith-Diagne

**Affiliations:** a Division of Mammals, Department of Vertebrate Zoology, Smithsonian National Museum of Natural History, Washington, DC, USA;; b Laboratories of Analytical Biology, Smithsonian National Museum of Natural History, Washington, DC, USA;; c African Aquatic Conservation Fund, Ngazobil, Senegal

**Keywords:** Atlantic humpback dolphin, mitogenome, Delphinidae, *Sousa*, Senegal

## Abstract

The Atlantic humpback dolphin remains an understudied, critically endangered cetacean species. Here, we describe the first complete mitogenome of *Sousa teuszii*, derived from an animal stranded on Île des Oiseaux, Sine Saloum, Senegal. The *S. teuszii* mitogenome is composed of 16,384 base pairs and is 98.1% identical to its closest relative with a mitogenome, *Sousa chinensis*. Phylogenetic analysis confirms its placement with *S. chinensis*, as well as the placement of the genus *Sousa* within subfamily Delphininae.

The Atlantic humpback dolphin (*Sousa teuszii*) is one of the world’s most critically endangered cetaceans (Collins et al. 2017). It is one of four currently recognized species in the genus *Sousa*, collectively known as ‘humpback dolphins’ (Jefferson and Rosenbaum 2014). *Sousa teuszii* is the westernmost species and is a resident of localized areas of shallow nearshore waters along the western coast of Africa from Western Sahara to Angola (Collins 2015). Estimates indicate a total population of 1500 mature individuals with increasing threats from human activities (Collins et al. 2017). The Senegal population has been estimated at a minimum of 103 individuals, the highest population estimation within the species range (Weir 2016). The species remains poorly understood, with few individuals sequenced for any molecular data (Frère et al. 2011; Mendez et al. 2013). Here we describe the first complete mitochondrial genome of *S. teuszii*.

The skin was collected from an adult female which stranded at Île des Oiseaux, Sine Saloum, Senegal (13°38′45.4′′N, 16°38′54.9′′W) on 8 May 2018. Skin samples were deposited along with a vouchered skull in the Division of Mammals of the Smithsonian National Museum of Natural History (NMNH) with the accession number USNM 605133. Total genomic DNA was extracted using a QIAGEN DNeasy Blood & Tissue Kit and sheared to an average fragment size of 500 bp using Covaris ME220. A whole-genome sequencing library was constructed using NEBNext Ultra II DNA Library Prep Kit for Illumina and sequenced on the Illumina MiSeq platform at NMNH with 150 bp paired-end read lengths.

Raw reads were trimmed using Trimmomatic (Bolger et al. 2014) and mapped to the mitogenome of the Indo-Pacific humpback dolphin (*Sousa chinensis*; NC_012057) using Geneious Prime 2019.3.1 (Biomatters Ltd., Auckland, New Zealand). A total of 142,263 paired-end reads mapped to the *S. chinensis* mitogenome with mean coverage of 1697×. The *S. teuszii* mitogenome (Genbank: MN365274) is 16,384 base pairs (bp) in length and contains the standard features present in a vertebrate mitogenome including 13 protein-coding genes, 2 rRNA genes, and 22 tRNA genes. We aligned the complete mitogenome of *S. teuszii* to 35 additional mitogenomes from 32 cetacean species using MAFFT (Katoh and Standley 2013). All 13 protein-coding genes, 2 rRNA genes, and the control region were included in a maximum-likelihood phylogenetic analysis using RAxML v.8 (Stamatakis 2014). All protein-coding genes were partitioned by codon position and added to three additional non-coding partitions (*12S*, *16S*, control region). The RAxML analysis was performed using an optimal partitioning scheme determined by PartitionFinder 2.0 (Lanfear et al. 2017), a GTR + GAMMA model for each partition, and 1000 bootstrap replicates using ‘rapid bootstrapping’.

*Sousa teuszii* is nested within Delphininae, and highly supported (100%) as the sister species of its congener *S. chinensis* (Figure 1). Based on pairwise comparisons, the *S. teuszii* mitogenome is 1.9% divergent from *S. chinensis* and ∼3% divergent from other delphinine dolphins. Results here mirror those of Horreo (2019); however, the mitogenomic phylogeny of Delphinidae differs significantly from nuclear phylogenies, which place *Sousa* in a more basal position within the subfamily Delphininae (McGowen et al. 2019).

**Figure 1.**
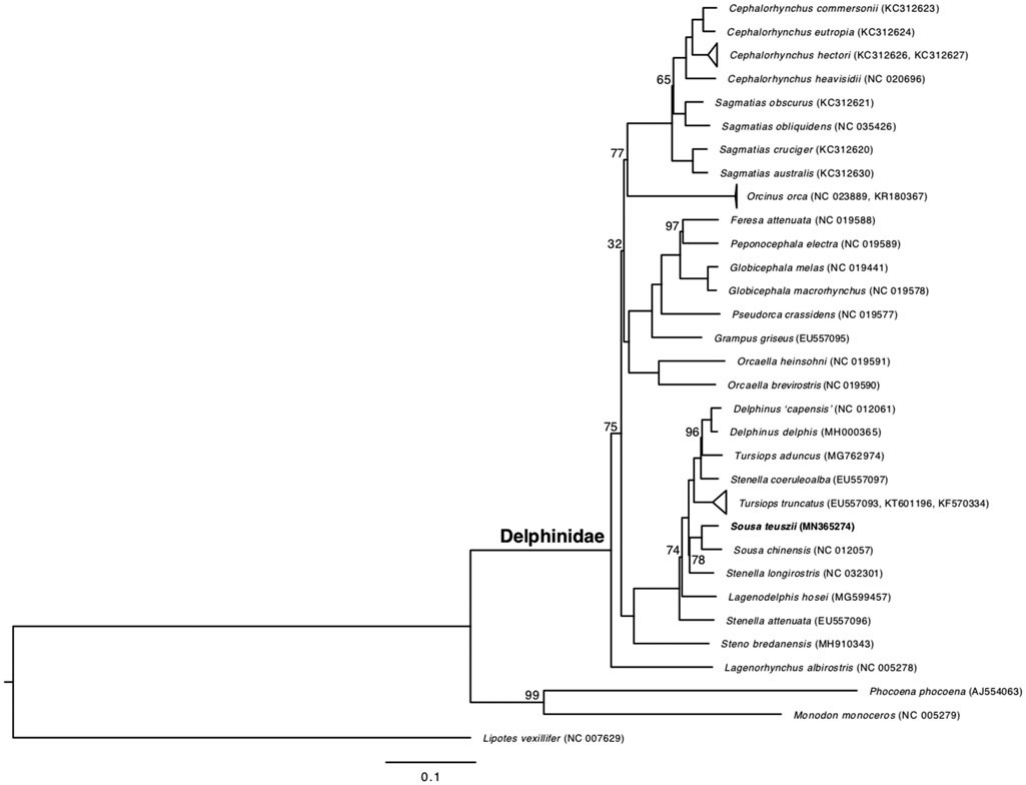
Maximum-likelihood phylogeny of delphinine dolphins and outgroups based on 13 protein-coding genes, *12S*, *16S*, and control region of 35 mitogenomes. Bootstrap values were 100 for each node except where noted over the corresponding branch.
